# Vitamin D supplements: a novel therapeutic approach for Alzheimer patients

**DOI:** 10.3389/fphar.2014.00006

**Published:** 2014-01-28

**Authors:** Cedric Annweiler, Spyridon N. Karras, Panagiotis Anagnostis, Olivier Beauchet

**Affiliations:** ^1^Department of Geriatric Medicine, UPRES EA 4638, University HospitalAngers, France; ^2^Robarts Research Institute, University of Western OntarioLondon, ON, Canada; ^3^Unit of Reproductive Endocrinology, First Department of Obstetrics and Gynecology, Medical School, Aristotle University of ThessalonikiThessaloniki, Greece

**Keywords:** vitamin D, neuroendocrinology, Alzheimer disease, dementia, research, multi-target drug, disease-modifying drug, memantine

## Introduction

Alzheimer disease (AD) is the main cause of dementia and loss of functional independence in the elderly (Aisen, 2010; Vellas and Aisen, 2010). AD is a chronic neurodegenerative disease characterized by a progressive decline of cognitive performance with a deleterious impact on social activities. AD is a worldwide big concern because of its adverse consequences and its expanding prevalence and incidence (Aisen, 2010; Vellas and Aisen, 2010). In order to reduce AD impact at an individual level and in terms of health and social costs, the development of efficient therapeutic strategies proves necessary. Regrettably, the development of all current specific medications is unsuccessful in clinical development phases (Birks, 2006; McShane et al., 2006; Anand et al., 2014). However, an interesting fact is that the prevalence of dementia in the general population might still be subject to changes. Indeed, a reduction in the prevalence of dementia is reported among English community-dwellers over the past two decades (Matthews et al., 2013). So, even if some factors, such as the increased life expectancy beyond 80 years, are unavoidable and augment the global prevalence of dementia, others may instead reduce its prevalence. For instance, efficient primary prevention of cardio-vascular disease is suspected to explain the reduced prevalence of dementia in England (Matthews et al., 2013). In the same vein, vitamin D, an “old” molecule primarily known for its effects on the bone, has been singled out in the last decade as an important biological component able to influence the natural history of AD. Here, we wish to highlight the difficulties experienced by the research on curative treatments and, in contrast, the prospects offered by multi-target drugs, especially those based on vitamin D.

## Treatments research in alzheimer disease: the irony of curative therapies

The only drugs currently available (i.e., cholinesterase inhibitors and memantine) slow down without changing the natural history of AD (Birks, 2006; McShane et al., 2006). Because of their symptomatic action, they are intended only for patients with mild-to-severe AD. Thus, one of the main AD challenges over the coming decade lies in the finding of a curative drug (Vellas and Aisen, 2010). Recent research has focused on “disease-modifying” medications to intervene in the pathogenesis of the disease, able to halt or slow the neurodegenerative process. To date, strategies to develop disease-modifying drugs have mainly targeted amyloid-β peptide (accumulation, aggregation, clearance) and/or the “tubulin associated unit” (TAU protein: phosphorylation and aggregation) by active and passive immunotherapy (Vellas and Aisen, 2010; Anand et al., 2014). Unfortunately candidate drugs, such as bapineuzumab, have failed in phase III clinical trials conducted in mild-to-moderate AD (Anand et al., 2014).

Because the earlier the disease-modifying treatment starts, the greater is the possibility of efficacy, it is important to initiate the treatment before the clinical manifestation of the overt disease. This is one of the reasons of the interest for the early diagnosis of AD. Importantly, this research has identified new morphological as well as functional and biological criteria that, combined with the classical neuropsychological expertise, help make the diagnosis of AD earlier and earlier (Schrötter et al., 2013). Such accuracy of the diagnosis of early-stage AD led in memory clinics to a drastic selection of participants for the curative clinical trials, with consequent substantial recruitment difficulties. The second consequence of this hyperselection was to address only a fraction of the patients followed in memory clinics, predominantly those with the prodromal stage of AD. For instance, the French National Centre for the Management of Trials on Healthcare Products (CeNGEPS), which involves more than ten university memory clinics (UMCs) in France, included in 2009 only 260 participants in clinical trials, although there were potentially 24,000 patients available in these centers. In other words, the curative drugs are addressing only 1.1% of AD patients followed in French UMCs. The question is then what can be offered to all other patients.

## Treatments research in alzheimer disease: the multi-target drugs

In addition to curative treatments, two other therapeutic approaches should be considered in AD. The first one is the non-drug-based approach, which is a multi-domain intervention designed to stimulate AD patients (de Sant'Anna and Morat, 2013). The second one is the multi-target drug approach, meaning that the treatment administered to AD patients simultaneously targets several neurodegenerative processes (Cavalli et al., 2008). The latter approach offers the opportunity to combine current standard anti-dementia symptomatic drugs with other neuroprotective agents to build a multi-regimen with multi-target effects. This questioning is central since patients at later stages of AD (i.e., with symptoms) could benefit from this new therapeutics, thus reducing chance inequalities between AD patients. In addition, it could benefit a larger number of patients and could be easily implemented compared with current trials, since it targets diagnosed patients who are already using a symptomatic treatment. Among the candidate molecules is the vitamin D, which has demonstrated in the last decade numerous neurological effects.

## Vitamin D and alzheimer disease: from an intriguing idea to a therapeutic option

About one billion people are deficient in vitamin D throughout the world. Older adults are especially affected, in particular those with AD with a prevalence of approximately 70–90% (Holick, 2007; Annweiler et al., 2011).

**Figure 1:**
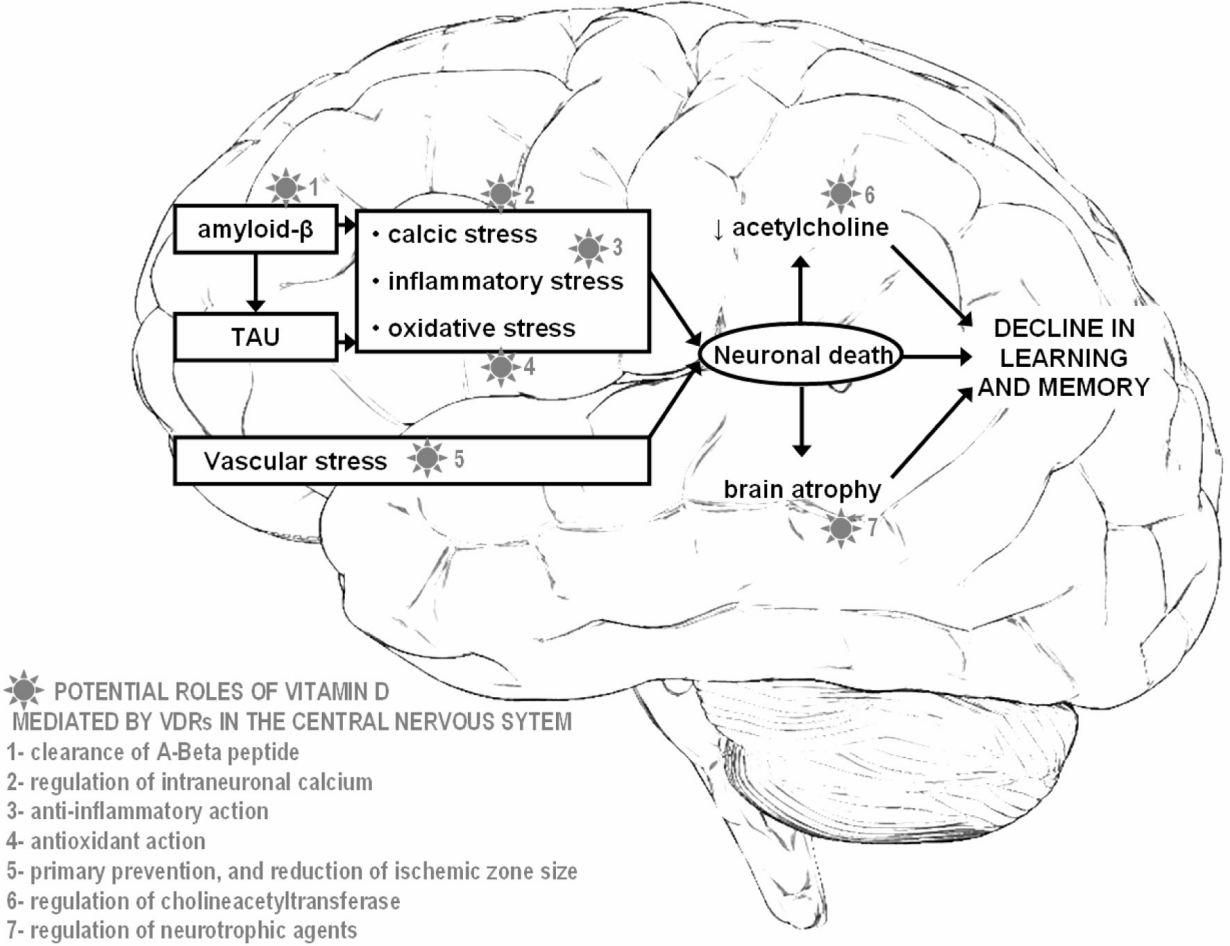
**Potential neuroprotective roles offered by vitamin D in Alzheimer disease**.

Besides its classical function of bone metabolism regulation, vitamin D has exhibited multiple biological targets mediated by its nuclear hormone receptor, the Vitamin D Receptor (VDR) (Kalueff and Tuohimaa, 2007; Annweiler et al., 2010). Specific actions on target organs such as the central nervous system (CNS) have been described, providing evidence for a neurosteroid action of vitamin D (Kalueff and Tuohimaa, 2007). In particular, VDRs are present in neurons and glial cells of the hippocampus, cortex and sub-cortex, all regions essential to cognition. The binding of vitamin D on the VDRs triggers neuronal protection against AD degenerative processes, including anti-inflammatory action, antioxidant effect, control of calcium homeostasis by regulating the concentration of intracellular calcium in hippocampal neurons, anti-atrophic effect by regulating neurotrophic agents, attenuation of Aß42 peptide accumulation by stimulating the phagocytosis of Aß peptide together with enhancing brain-to-blood Aß efflux transport at the blood-brain barrier, and the prevention of acetylcholine defect by increasing the activity of choline acetyltransferase (thus the bioavailability of acetylcholine) in the brain (Kalueff and Tuohimaa, 2007; Annweiler et al., 2010; Annweiler and Beauchet, 2011). These experimentally-described neuroprotective properties of vitamin D may help, in the case of normalized vitamin D status, to address against the decline of brain function in AD, especially against cognitive decline (Figure 1).

Epidemiology is consistent with this notion, and has repeatedly shown that low serum concentrations of vitamin D are cross-sectionally associated with global cognitive impairment (Annweiler et al., 2009), the people with hypovitaminosis D having more cognitive disorders (Etgen et al., 2012), specifically more executive dysfunctions (Annweiler et al., 2013a). Specifically, it has been reported that people with AD have lower vitamin D status than controls (Annweiler et al., 2013b). They also exhibit increased levels of vitamin D-binding protein (Moon et al., 2013) and lower levels of 25-hydroxyvitamin D in the cerebrospinal fluid (Johansson et al., 2013) compared to controls. Longitudinal prospective studies have enabled to better understand this association and have reported a temporal sequence, with hypovitaminosis D preceding the onset of cognitive decline (Llewellyn et al., 2010).

Recent biomedical literature further suggests that the VDRs may confer genetic risk for AD. Some human variants appear less sensitive to vitamin D and more likely to experience cognitive decline. For instance, AD patients have reduced levels of VDR mRNA (Sutherland et al., 1992). In contrast, it has been reported that the overexpression of VDR may suppress amyloid precursor protein transcription (Wang et al., 2012). A significant association has also been shown between the VDR gene *APA1* polymorphism and the onset of AD, the *Aa* genotype multiplying by 2.3 the risk of AD compared to the *AA* genotype (Gezen-Ak et al., 2007).

It seems thus crucial to maintain high vitamin D levels in the elderly, especially in those with AD. In line with this, high intake of vitamin D (whether from food, supplements, or sun exposure) is associated with better cognitive function in older individuals. Having more than 800UI of vitamin D supplies per day divides the risk of AD by 5 after 7 years of follow-up (Annweiler et al., 2012a). This neuroprotective effect has been confirmed by clinical comparative trials reporting cognitive improvement after vitamin D supplementation whether in general aged population (Prybelski et al., 2008; Annweiler et al., 2012b) or in patients who already have symptoms of AD (Stein et al., 2011). The cognitive benefits of supplementation appear at 4 weeks (Prybelski et al., 2008), and particularly for executive functions and processing speed (Annweiler et al., 2013a).

It has to be noted yet that, although most of older adults have hypovitaminosis D, not all develop AD. It is thus unlikely that hypovitaminosis D alone explains the onset of AD, and that vitamin D supplementation is sufficient to prevent AD by itself. One solution might thus be to combine vitamin D with a symptomatic anti-dementia treatment to build a multi-target drug (Annweiler and Beauchet, 2012). Indeed, if hypovitaminosis D explains in part the pathological process of AD, it may also enhance the effectiveness of standard anti-dementia treatments or account at least partially for the resistance to these treatments. Even speculative, this engages clinicians to replenish vitamin D before starting anti-dementia treatments or to use vitamin D as an adjunct to standard treatments. In line with this, a recent 6-month controlled trial has recently reported that the combination of memantine + vitamin D was superior to memantine alone and vitamin D alone in preventing cognitive decline among AD participants (Annweiler et al., 2012c). In fact, those taking both molecules had a clinically relevant and statistically significant gain of 4 points on the Mini-Mental State Examination. These results were consistent with an *in vitro* experiment, which showed that cortical axons degenerate less after exposure to amyloid-β peptide or glutamate in microfluidic neuronal cultures enriched with memantine plus vitamin D compared to control medium and compared to cultures enriched with memantine only or with vitamin D only (Annweiler et al., 2014).

## Conclusions

In conclusion, AD is a public health concern due to its very high prevalence and because the few drugs currently available are only symptomatic. Current therapeutic research, which focuses mainly on curative disease-modifying treatments, addresses a very small fraction of AD patients, predominantly those with prodromal disease and no symptoms of dementia. It is thus necessary to consider the patients who already suffer from moderate-to-severe AD, who constitute the majority of AD patients. One of the best hopes for them may rely on the development of multi-target drugs. In particular, using vitamin D supplements as an adjunct to standard anti-dementia treatments appears to be a simple, inexpensive, and efficient therapeutic strategy and leads to encouraging prospects for the correction of neurological disorders in AD.

## Authors contributions

All authors meet all of the following criteria: (1) Contributing to the conception and design, or analyzing and interpreting data; (2) Drafting the article or revising it critically for important intellectual content; and (3) Approving the final version to be published.

### Conflict of interest statement

The concept of combining memantine with vitamin D in the prevention and treatment of Alzheimer's disease and related disorders was patented by Angers University Hospital and the University of Angers. The authors report no conflicts of interest with this research. None of the authors have a personal financial interest in this research.
